# Analysis of the human diseasome using phenotype similarity between common, genetic, and infectious diseases

**DOI:** 10.1038/srep10888

**Published:** 2015-06-08

**Authors:** Robert Hoehndorf, Paul N. Schofield, Georgios V. Gkoutos

**Affiliations:** 1 Computational Bioscience Research Center, King Abdullah University of Science and Technology, 4700 King Abdullah University of Science and Technology, Thuwal 23955-6900, Kingdom of Saudi Arabia; 2 Computer, Electrical and Mathematical Sciences & Engineering Division, King Abdullah University of Science and Technology, 4700 King Abdullah University of Science and Technology, Thuwal 23955-6900, Kingdom of Saudi Arabia; 3 Department of Physiology, Development & Neuroscience, University of Cambridge, Downing Street, Cambridge, CB2 3EG, UK; 4 Department of Computer Science, Aberystwyth University, Llandinam Building, Aberystwyth, SY23 3DB, UK

## Abstract

Phenotypes are the observable characteristics of an organism arising from its response to the environment. Phenotypes associated with engineered and natural genetic variation are widely recorded using phenotype ontologies in model organisms, as are signs and symptoms of human Mendelian diseases in databases such as OMIM and Orphanet. Exploiting these resources, several computational methods have been developed for integration and analysis of phenotype data to identify the genetic etiology of diseases or suggest plausible interventions. A similar resource would be highly useful not only for rare and Mendelian diseases, but also for common, complex and infectious diseases. We apply a semantic text-mining approach to identify the phenotypes (signs and symptoms) associated with over 6,000 diseases. We evaluate our text-mined phenotypes by demonstrating that they can correctly identify known disease-associated genes in mice and humans with high accuracy. Using a phenotypic similarity measure, we generate a human disease network in which diseases that have similar signs and symptoms cluster together, and we use this network to identify closely related diseases based on common etiological, anatomical as well as physiological underpinnings.

Over the last decade, the rapid emergence of new technologies has redefined our understanding of the genetic and molecular mechanisms underlying disease. For example, we can now identify genetic predisposition to diseases, and responses to environmental factors, through a rapidly increasing number of genome-wide association studies. These studies utilize genetic variation in human populations to identify sequence variants that predispose some individuals to common or complex diseases. Such studies also reveal a variety of differences between disease manifestations. Application of sequencing technologies to disease studies has been particularly successful for genetically-based diseases. For example, full exome sequencing is an approach that has emerged to identify causative mutations underlying congenital diseases, and is successfully applied widely^1^,^2^. In contrast to genetically based diseases, the investigation of infectious diseases poses an additional challenge as it requires not only the understanding of the physiology and patho-physiology of a single organism, but the investigation of two or more organisms, their interactions, and the response of one organism to the other. Similarly, investigations of environmentally-based diseases require understanding the response of organisms to environmental influences such as chemicals, radiation, habitat or society.

For each disease class (genetically-based, environmental, and infectious), the genetic architecture of an organism plays a vital role in the disease manifestation it exhibits, including severity of symptoms, complications, as well as its response to therapeutic agents. A key to gaining an in-depth understanding of the molecular basis of disease is the understanding of the complex relationship between the genotype of an organism and the phenotypic manifestations it exhibits in response to certain influences (genetic, environmental, or exposure to an infectious agent). To achieve such a goal, it is imperative that there is a consistent and thorough account of the various phenotypes (including signs and symptoms) exhibited by an organism in response to etiological influences.

To utilize phenotype data for disease studies, information about Mendelian diseases has been historically well documented in various formats and, more recently, in electronic resources such as the Online Mendelian Inheritance in Man (OMIM)^3^ database and the Orphanet^4^ resource. Both OMIM and Orphanet provide a catalog of human genes and genetic disorders, and contain a variety of textual information including patient symptoms and signs. Ontologies (i.e., structured, controlled vocabularies that formally describe the kinds of entities within a domain) such as the Human Phenotype Ontology (HPO)^5^ have been created in an attempt to provide a comprehensive controlled vocabulary and knowledge base describing the manifestations of human diseases, and these ontologies have been applied to characterize diseases in the OMIM and Orphanet databases^6^,^7^. Additionally, ontology-based analysis of phenotype data has also been shown to significantly improve the accuracy of finding disease gene candidates from GWAS data^8^ and assignation of phenotypes to genes in Copy Number Variation syndromes^9^.

The remarkable conservation of phenotypic manifestations across vertebrates implies a high degree of functional conservation of the genes participating in the underlying physiological pathways. Our increasing ability to identify such functions as well as their role in human disease using a variety of organisms and approaches, such as forward and reverse genetics, renders animal models valuable tools for the investigation of gene function and the study of human disease. Phenotype information related to model organisms is also being described using ontologies such as the Mammalian Phenotype Ontology (MP)^10^, and data annotated with these ontologies is being systematically collected and organized in model organism databases^11^. The systematic coding of phenotypic and molecular information related to humans and other model species facilitates integrative approaches for identifying novel disease-related molecular information^7^,^12^,^13^, prioritizing candidate genes for diseases based on comparing the similarity between animal model phenotypes and human disease phenotypes^14^,^15^ as well as predicting novel drug-target interactions, drug targets and indications^16^,^17^,^18^,^19^.

Extension of these strategies and tools for the study of common and infectious diseases has been hampered by the lack of an infrastructure providing phenotypes associated with common and infectious diseases, and integrating this information with the large volumes of experimentally verified and manually curated data available from model organisms. We have now generated a resource of disease-associated phenotypes for over 6,000 common, rare, infectious and Mendelian diseases. The phenotypes and diseases are characterized using ontologies and interoperate with widely used ontologies used for describing human and model organism phenotypes^20^. We evaluate our resource of disease-associated phenotypes against its ability to identify genes for human diseases, and demonstrate that our method yields disease phenotypes that are comparable to those available from OMIM when applied to finding candidate genes for Mendelian diseases. We further demonstrate the potential applications of our resource by revealing closely related diseases based on common etiological, anatomical as well as physiological underpinnings. We make our results freely available at http://aber-owl.net/aber-owl/diseasephenotypes/ and provide a visualisation environment for them at http://aber-owl.net/aber-owl/diseasephenotypes/network/.

## Results

We have created a resource of disease-associated phenotypes for diseases in the Human Disease Ontology (DO)^21^. For this purpose, we have identified co-occurrences between names of diseases (from DO) and names of phenotypes from the Human Phenotype Ontology (HPO)^5^ and the Mammalian Phenotype Ontology (MP)^10^ in abstracts and titles of 5 million articles in Medline.

We employ several different scoring functions to rank the co-occurrences based on their significance within our corpus. In particular, we use the normalized pointwise mutual information (NPMI), T-Score, Z-Score and the Lexicographer’s mutual information scores^22^ to rank the co-occurrences. The phenotypes associated with diseases, scored by our scoring functions, can be viewed and downloaded at http://aber-owl.net/aber-owl/diseasephenotypes.

As our scoring functions associate a value with each identified co-occurrence between a term referring to a disease class and a term referring to a phenotype class, we use known gene-disease associations from the OMIM database to identify a cutoff that maximizes the potential to prioritize candidate genes of disease based on phenotypic similarity. For this purpose, we use the PhenomeNET system^14^ to systematically compute the phenotypic similarity between disease phenotypes and mouse model phenotypes, and we compare the results against known mouse models of disease from the Mouse Genome Informatics (MGI) database^11^, as well as, using human-mouse orthology, to known gene-disease associations in the OMIM database. We quantify the predictive power of the phenotype by computing the area under the ROC curve for predicting gene-disease associations through phenotype similarity.

**Figure 1:**
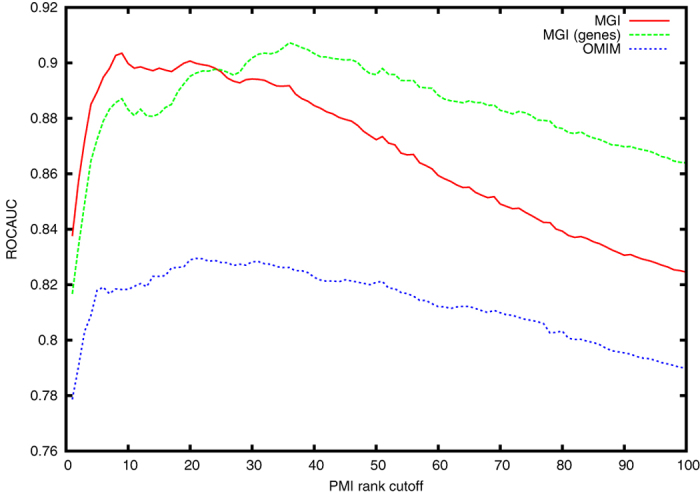
ROCAUC values obtained when using different cutoffs for the rank of the pointwise mututal information co-occurrence measure. Based on three different evaluation datasets, we find that the top-ranking 9 phenotypes maximize ROCAUC when comparing against mouse disease models in MGI (ROCAUC 0.903* *± 0.015), 21 when comparing against OMIM’s gene-disease associations (ROCAUC 0.829* *± 0.014), and 36 when comparing against genes involved in a disease in MGI (ROCAUC 0.907* *± 0.012).

To standardize the number of phenotypes associated with a disease, we rank all phenotype-disease associations for each disease by their normalized pointwise mutual information score. We then utilize an increasing number of phenotypes for each disease, compute their similarity to mouse model phenotypes, compare the ranked list of mouse model–disease similarities to known mouse models of diseases, and use the ROCAUC in retrieving these known mouse models to identify the optimal cutoff in the number of phenotype annotations for each disease. When considering the first *n* ranks of phenotypes, we exclude all diseases from the analysis for which the normalized pointwise mutual information has no positive value at rank *n*. Figure 1 shows the resulting ROCAUC for varying cutoff values. We find that the optimal choice of the number of disease–phenotype associations (i.e., the number that maximizes their performance in predicting candidate genes involved in the disease) depends on our evaluation dataset: 9 when comparing against mouse disease models in MGI (ROCAUC 0.903 ± 0.015), 21 when comparing against OMIM’s gene-disease associations (ROCAUC 0.829 ± 0.014), and 36 when comparing against genes involved in a disease in MGI (ROCAUC 0.907 ± 0.012). This divergence is likely a result of a sensitivity of our similarity measure to the number of phenotypes that is compared. We use the optimal value for the OMIM evaluation set as the main cutoff in the remaining analysis. Using this cutoff, the ROCAUC values are 0.900 ± 0.012 for MGI’s disease models and 0.896 ± 0.015 for genes involved in a disease in MGI, and we have mined phenotypes for 6,220 disease classes in DO, using a total of 9,646 different phenotype classes from the HPO and MP (6,000 from HPO and 3,646 from MP). With 21 phenotypes for each disease, we associate almost double the number of phenotypes as currently in the HPO database for OMIM disease which associates 11.1 phenotypes on average to each of the 6,470 OMIM entries it contains.

**Figure 2:**
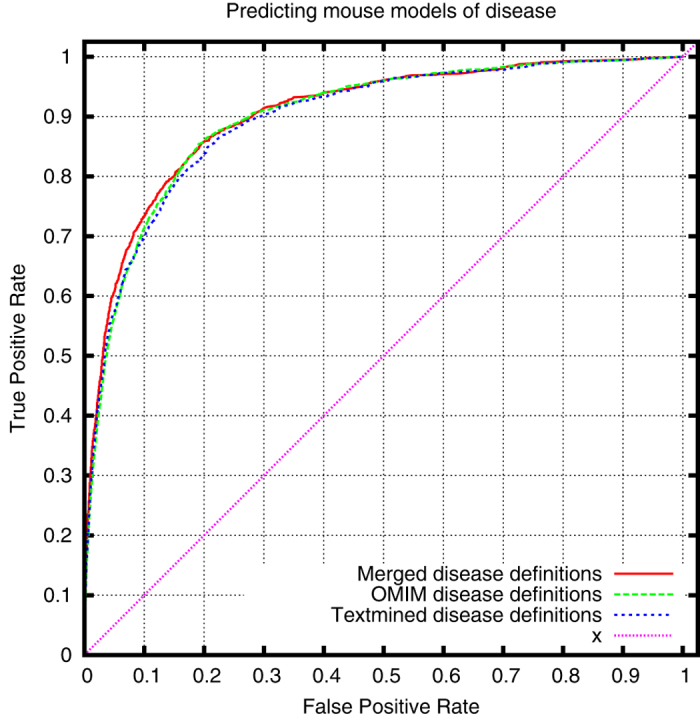
ROC curve for cross-species prioritization of disease models using MGI’s gene-disease association dataset and merging the phenotypes for genotypes affecting single genes (ROCAUC: 0.896 ± .015 (text-mined), 0.904 ± .018 (OMIM), 0.904 ± .015 (merged)).

**Figure 3:**
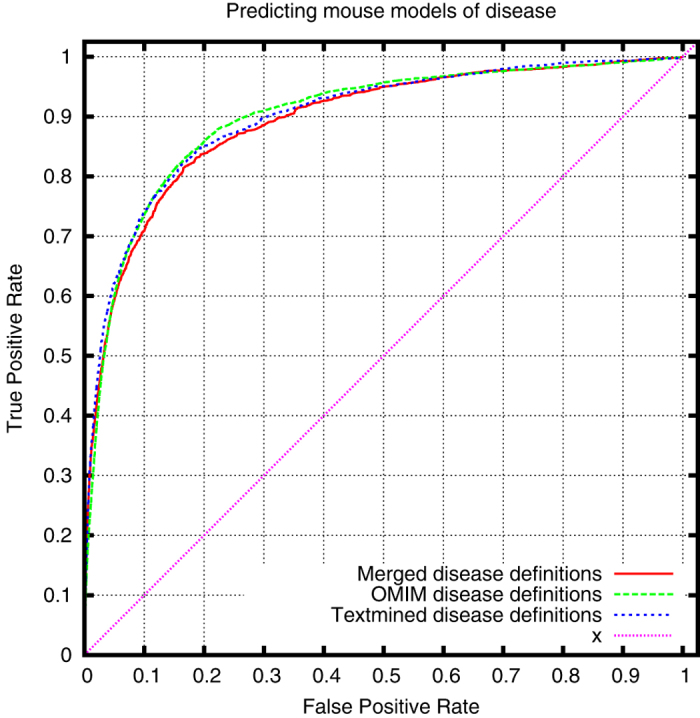
ROC curve for cross-species prioritization of disease models using MGI’s genotype-disease association dataset (ROCAUC: 0.900 ± 0.012 (text-mined), 0.900 ± 0.012 (OMIM), 0.893 ± 0.013 (merged)).

**Figure 4:**
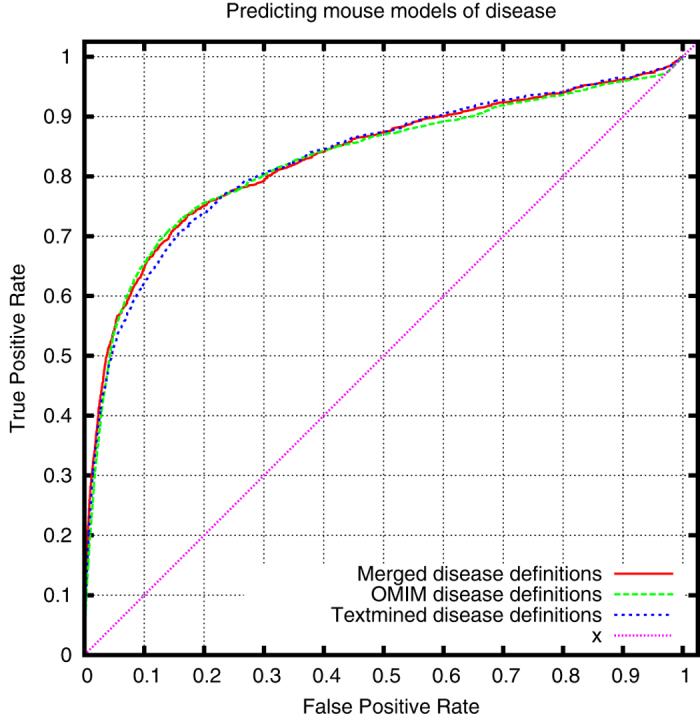
ROC curve for cross-species prioritization of disease models using OMIM MorbidMap’s gene-disease association dataset (ROCAUC: 0.823 ± 0.015 (text-mined), 0.828 ± 0.011 (OMIM), 0.832 ± 0.014 (merged)).

Using only heritable diseases from OMIM, we can demonstrate that our text-mined phenotypes come close to the phenotypes associated with OMIM diseases in the HPO database when applied to prioritizing candidate genes of disease. Figures 2, 3 and 4 show the comparison of the performance of our text-mined phenotypes with the original OMIM phenotypes in PhenomeNET. To further test our phenotypes, we have merged the original OMIM phenotypes with our text-mined phenotypes, and we can demonstrate a small increase in ROCAUC over both our text-mined phenotypes and the original OMIM phenotypes (except when using MGI’s genotype-disease associations as evaluation dataset).

**Figure 5:**
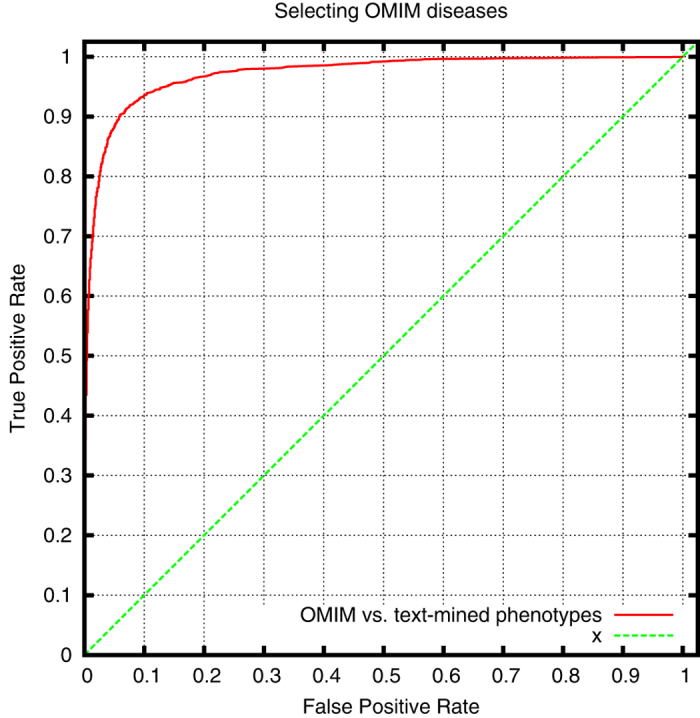
ROC curve for ranked retrieval of OMIM diseases by semantic similarity to text-mined disease phenotypes (ROCAUC: 0.972 ± 0.008).

We further evaluate the overlap with OMIM disease definitions, as characterized by the HPO database. We use two measures to quantify the overlap. First, we directly compute the set overlap (Jaccard index) between the HPO phenotypes we have text-mined for each disease and the HPO phenotypes associated with the disease in the HPO database. The average Jaccard index between our disease definitions and the corresponding OMIM diseases is 0.074 (0.315 when considering the phenotypes together with all their superclasses). We also compute the percentage of coverage of the OMIM phenotypes in our disease definitions. Using our text-mining approach, we cover on average 23.5% of the phenotypes in OMIM (55.7% when considering the phenotypes together with all their superclasses). Finally, we compute the semantic similarity between our text-mined disease definitions and the phenotypes associated with the disease in HPO, and use ROC analysis to quantify the performance of directly identifying a matching disease. Figure 5 shows the resulting ROC curve with a ROCAUC of 0.972 ± 0.008.

Since gene–disease associations are only available for genetically based diseases, we further evaluate the disease phenotypes by determining whether the same drugs are used to treat phenotypically similar diseases. For this purpose, we compute the phenotypic similarity between all diseases, and identify drugs and their indications in the SIDER database^23^. Within the disease–disease similarity matrix consisting of all diseases, the probability that a randomly chosen pair of diseases that is treated with the same drug is ranked higher than a randomly chosen pair of diseases that is not treated with the same drug is 0.647 ± 0.005.

**Figure 6:**
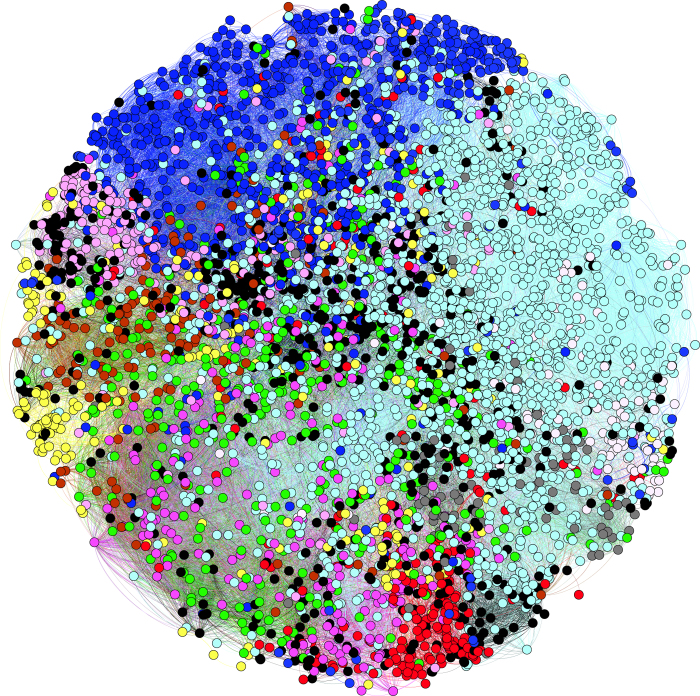
An overview over the disease–disease similarity network generated by our approach as well as six disease modules obtained by filtering for disease categories in DO. The graph is based on a force-directed layout using the similarity between diseases as attraction force. Nodes are colored according to the top-level DO category in which they fall: cyan–disease of cellular proliferation, blue–nervous system and mental disease, red–cardiovascular disease, yellow–metabolic disease, green–infectious disease, magenta–immune system disease, brown–integumentary disease, pink–muscoloskeletal disease, gray–urinary system disease.

Using the phenotypes associated with DO diseases, we compute a pairwise disease–disease similarity based on semantic similarity of their associated phenotypes. From the resulting similarity matrix, we generate a disease–disease network based on phenotypes from the top-ranking 0.5% of disease–disease similarity values (i.e., *p *≤ 0.005 within the distribution of disease–disease similarity values). The generated network consists of 5,030 nodes and 65,795 edges, and is shown in Fig. 6. In the network, 16 connected components exists, the largest one with 4,991 nodes and the remaining components with between two and five nodes. The average node degree is 26.2. For each disease, we also identify top-level DO categories, and assign node attributes in the network based on the DO categories in which a disease falls. The disease–disease similarity network can be accessed online at http://aber-owl.net/aber-owl/diseasephenotypes/network.

We also use disease–disease similarity to compute phenotypic coherence of diseases within their respective disease category. For this purpose, for each disease, we sort all other diseases based on their phenotypic similarity, and identify the ranks at which other diseases in the same category appear. The results (summarized in Table 1) are ROCAUC values for each of DO’s top-level categories that quantify how phenotypically similar are the diseases within the same category.

Finally, we use the FLAME clustering algorithm^24^ to identify clusters within the disease–disease similarity matrix. FLAME generates 198 clusters with an average purity of 0.575. We further compare the resulting clusters against the classification of diseases provided by the top-level categories of DO. The Rand index^25^ (a measure of similarity between two clusterings) between the FLAME clusters and the top-level DO grouping is 0.828.

## Discussion

### Related work

Associations between phenotypes, signs and symptoms on one side and diseases on the other have been used to gain insights into the modular nature and network structure of human diseases and drug indications^12^,^26^,^27^. In prior work, text-mining based on labels of diseases and labels of phenotypes (signs and symptoms)^27^, or the identifiers of the Medical Subject Headings (MeSH) Thesaurus^28^ that are associated with article citations in Pubmed, have been used to identify associations between disease and phenotype. In general, the resulting disease–phenotype associations have been evaluated based on their ability to reveal or explain perceived clusters of diseases^12^, or group diseases with known common etiology together^26^,^27^, based on gold standard comparison and clustering for common drug targets^27^.

One fundamental question that has not been answered by any of the prior approaches has been what kind of evidence or support would be required to consider a disease–phenotype association as “correct”. This is a fundamental challenge in any kind of phenotype- or symptom-based characterization of disease. Most diseases have cardinal signs and symptoms which will always be associated with a disease. However, a large number of signs and symptoms for a disease are not always present but rather occur with varying frequency, and even very rare manifestations may prove to be highly useful in the context of differential diagnosis. In our evaluation, we provide a quantifiable measure through comparison against experimental data which can be used to determine–and maximize–the performance of our text-mined disease–phenotype associations in predicting candidate genes for human disease. We therefore provide an objective measure that can be used to determine how applicable a set of disease–phenotype associations are to a particular scientific question–in our case, identifying candidate genes for diseases of genetic origin. While different applications may require different sets of phenotypes associated with a disease, we believe that this evaluation strategy also provides an indication of the potential utility of these text-mined phenotypes for further scientific investigations, i.e., how these disease–phenotypes associations can be used to support additional studies about the mechanisms underlying diseases.

One main limitation of our evaluation is that it is limited to the genetic diseases in OMIM, while the majority of diseases in the DO are complex, common or acquired through the effects of environment or infectious agents. Other approaches, such as clustering diseases based on similarity and identifying meaningful, well-known clusters^12^,^26^, or comparison with known drug indications^27^, can evaluate the biological validity of generated associations, but often cannot quantify the results.

### Novel candidate genes based on text-mined phenotypes

Through our approach, we not only obtain phenotypic characterization of common and infectious diseases, but we have also obtained novel phenotype associations for genetically-based diseases in OMIM for which currently no phenotypic characterization exists either in the HPO annotations or as a clinical synopsis in OMIM.

The HPO database contains phenotype annotations for 9,286 OMIM entries (genes and diseases). Through the DO–OMIM mappings and our method, we obtain phenotypes for 1,683 OMIM entries, 115 of which have no phenotype annotations in HPO or an associated clinical synopsis in OMIM. For example, *Halo Nevi* (*Leukoderma acquisitum centrifugum of Sutton*, OMIM:234300), a dermatological condition in which melanocytes are destroyed by CD8+ cytotoxic T lymphocytes^29^, has currently no clinical synposis in OMIM and consequently no associated phenotypes in the HPO database, while we identify several phenotypes, including *variable depigmentation* (MP:0010016), *abnormal melanocyte morphology* (MP:0002877) and *Vitiligo* (HP:0001045) as phenotypes, all of which are known to be associated with halo nevi^30^.

**Figure 7:**
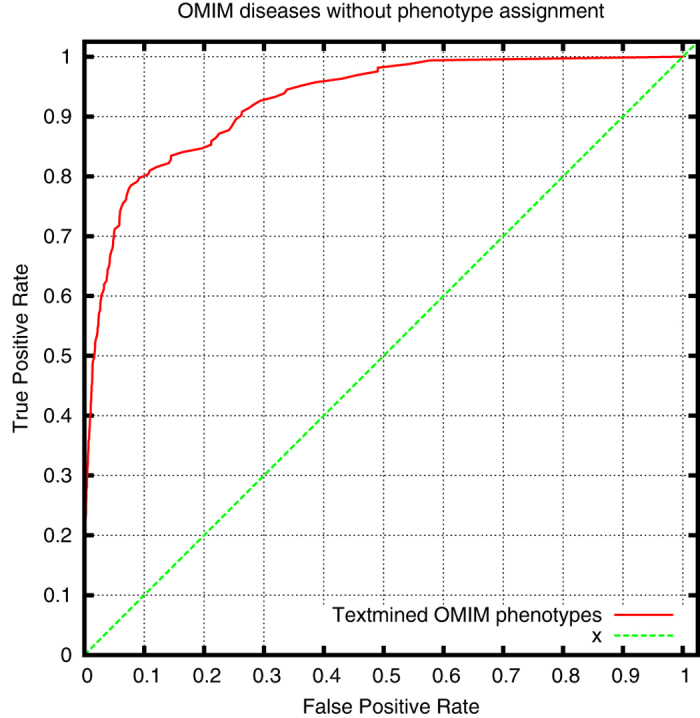
ROC curve for ranked retrieval of MGI disease models by semantic similarity to text-mined phenotypes of diseases without clinical synopsis in OMIM (ROCAUC: 0.926 ± 0.049).

For these 115 diseases, 167 disease models are known in the mouse. We can prioritize the correct model with ROCAUC of 0.926 ± 0.049 for this set of 115 diseases (Fig. 7).

### Exploring disease–disease similarities

The disease–disease similarity network (Fig. 6) shows phenotypic similarity relationships between common, genetic, infectious and environmental diseases. Each node in the network represents a disease and is colored according to its corresponding top-level disease class in DO. Based on this similarity network, we observe that diseases of different systems and pathological processes can be separated on the basis of phenotypic relatedness. DO classifies both by anatomical site or system, and by general pathology, and for each of the classifications, despite these different criteria, we find that diseases within one category are usually in close proximity to each other on the basis of phenotypic relatedness alone. This is supported by our cluster analysis, in which we find a strong similarity between clusters produced based on phenotypic similarity and top-level distinctions made by DO (Rand index 0.828).

**Figure 8:**
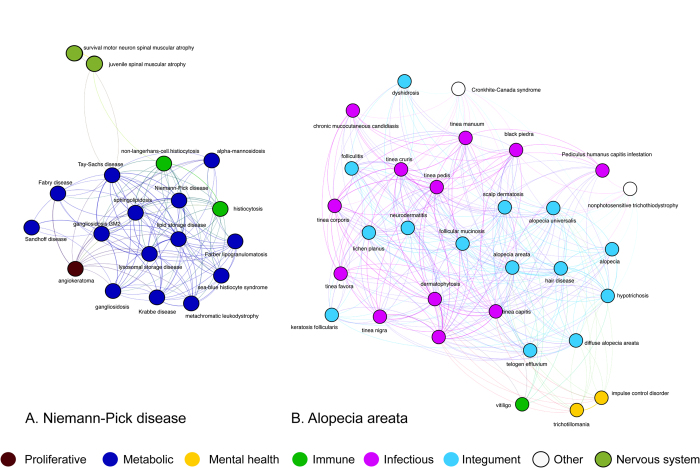
Left: The sub-network around Tay-Sachs disease, showing a range of lysosomal storage diseases.**Right**: Dermatological and other disorders phenotypically similar to *Alopecia areata*.

We can also identify similarity between etiologically related disease groups which show overlapping phenotypes. One example of these groups are the lysosomal storage diseases. All cells contain lysosomes which contain soluble acid hydrolases whose role is to process a wide range of substrates. Failure to perform this function results in lysosomal accumulation of unmetabolized proteins lipids and carbohydrates, which are the primary cause of disease through their effects on cellular metabolism. The pathways by which these accumulations exert their pathological effects are only just becoming understood, but they display an extensive range of disease symptoms with central neurological involvement and a wide range of peripheral phenotypes with very variable individual manifestation^31^. Figure 8 shows the phenotypic relationships between the sphingolipidoses, bringing together Niemann-Pick, Gaucher’s and Tay-Sachs diseases along with the leukodystrophies, metachromatic leukodystrophy and Krabbe disease, Sea-Blue histocyte syndrome and Farber disease. Interestingly, we find two forms of spinal muscular atrophy in the phenotypic neighborhood of these diseases which are not generally considered to be caused by a lysosomal storage disorder. However, it has recently been reported that in *FIG4*-deficient individuals (Charcot-Marie-Tooth disease type 4J), who show spinal motor neuron degeneration, muscular weakness and atrophy, neurons and glial cells accumulate lipid and protein, reminiscent of Niemann-Pick, Tay-Sachs and type IV mucolipidosis. This pattern of accumulation is associated with neuronal degeneration and demyelination of peripheral nerves in the GM2 gangliosidoses^32^,^33^ and likely to be the case of the neuropathy in Charcot-Marie-Tooth disease type 4J. The inclusion of the spinal muscular atrophies in the sphingolipidosis phenotypic neighbourhood is therefore likely to be due to the impact of lysosomal storage disorders of the motor system which are manifested as neurogenic muscular atrophy. This striking phenotypic similarity is similar in type to that seen in the ciliopathies^34^ where a range of related phenotypes reflect lesions in a collection of molecules involved in different aspects of cilium assembly or function, which, along with other examples, lead Oti and Brunner^35^ to postulate the existance of common functional modules underlying the phenotypic profiles of diseases.

The integumentary diseases (Fig. 8) also form a group of diseases with high phenotypic similarity and with clear demarcation between different types of disease. For example, alopecia, telogen effluvium, alopecia areata, alopecia universalis and follicular mucinosis, all of which are diseases involving hair follicles and causing hair loss, are found in close phenotypic proximity to each together. Interestingly, this region contains trichotillomania–the obsessive plucking of hair–and Cronkhite-Canada syndrome, a recently recognized sporadic syndrome comprising gastrointestinal hamartomatous polyposis, and the dermatological triad of alopecia, onychodystrophy, and hyperpigmentation^36^. The inclusion of keratosis follicularis characterized by follicular hyperkeratosis and progressive cicatricial alopecia in this phenotypic neighbourhood similarly shows the richness of the phenotypic data collected by text mining.

Unsurprisingly, we also find (see Table 1) that diseases classified by anatomical site or system (e.g., reproductive system diseases, respiratory diseases) exhibit higher phenotypic homogeneity than diseases classified by their pathological mechanism (e.g., infectious diseases, genetic diseases). In particular, we observe that narrowly defined disease categories such as *reproductive system disease*, *respiratory system disease* or *urinary system disease* exhibit a high phenotypic homogeneity; broad categories such as all the infectious diseases or diseases of cellular proliferation, on the other hand, are relatively heterogeneous. However, all of DO’s top-level categories cluster significantly based on their phenotypic similarity, and diseases falling into more specific DO categories (such as lysosomal storage diseases) cluster closely as well, demonstrating that not only Mendelian diseases form disease modules^12^,^35^, but also common diseases.

## Conclusions

Exploring diseases through their associated phenotypes has major applications for biomedical research, and several studies have primarily relied on disease phenotypes to reveal functional disease modules^12^,^26^,^35^, candidate genes of disease^14^,^37^, prioritize genes in GWAS studies^38^, and investigate drug targets and indications^17^,^18^,^39^,^40^. While the majority of these investigations have been focused on genetic diseases, application of similar methods may lead to novel insights into the patho-biology of common and infectious diseases as well.

## Materials and Methods

### Ontologies and vocabularies

We use the Human Phenotype Ontology (HPO)^5^ and the Mammalian Phenotype Ontology (MP)^10^ as vocabularies that provide terms referring to phenotypes, signs and symptoms associated with diseases. Additionally, the MP is used to describe mouse model phenotypes^41^, and we rely on comparison to mouse model phenotypes for the evaluation of our approach.

We use the Human Disease Ontology (DO)^21^ as an ontology of diseases. The DO contains a rich classification of rare and common diseases, and spans heritable, developmental, infectious and environmental diseases. All ontologies were downloaded from the OBO Foundry website^42^ on 2 July 2013.

### Semantic mining with Aber-OWL: Pubmed

We make use of the Aber-OWL: Pubmed infrastructure to semantically mine Medline abstracts. Aber-OWL: Pubmed (http://aber-owl.net/aber-owl/pubmed/) consists of an ontology repository, a reasoning infrastructure capable of performing OWL-EL reasoning over the ontologies in the repository, a fulltext index of all Medline 2014 titles and abstracts as well as all Pubmed Central articles, and a search interface. Aber-OWL: Pubmed uses an Apache Lucene (http://lucene.apache.org) index to store the articles. Before indexing, every text is processed using Apache Lucene’s English language standard analyzer which tokenizes the text, normalizes text to lower case, and applies a list of stop words.

To identify documents which contain references to a disease or phenotype term, we first limit our search to Medline abstracts and treat documents as consisting of a title and the abstract. We then limit our corpus to documents in which at least one term from a phenotype ontology (HPO or MP) or the DO occurs. As a result of this filtering step, we use a corpus consisting of 5,164,316 documents.

We use the information in ontologies together with the Aber-OWL reasoning infrastructure to identify the set of terms referring to a disease or phenotype. For this purpose, we first identify all labels and synonyms *Lab*(*C*) associated with a class *C* in an ontology. We then define the set of terms *Terms*(*C*) referring to a class *C* as:


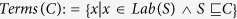


According to this definitions, the set *Terms*(*C*) refers to the set of labels and synonyms of *C* or any subclass of *C*, as inferred using the automated reasoner employed by the Aber-OWL infrastructure.

To identify the number of documents in which a disease or phenotype term occurs, we construct a Lucene query based on *Terms*(*D*) and *Terms*(*P*) in which we concatenate each member of *Terms*(*D*) or *Terms*(*P*) using the OR operator: 
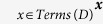
 and 
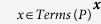
. As a result, the Lucene query will match any document (title or abstract) that contains a label or synonym of a class *D* or *P*. To identify the number of documents in which *D* and *P* occur together, we concatenate both queries using the AND operator: 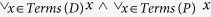.

We use *Docs*(*q*) to refer to the set of documents satisfying the query *q*, *n*_*D*_ to refer to the number of documents in which a term referring to disease *D* occurs, *n*_*P*_ to refer to the number of documents in which a term referring to a phenotype *P* occurs, and *n*_*DP*_ to refer to the number of documents in which both a term referring to *D* and a term referring to *P* occurs:


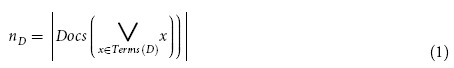



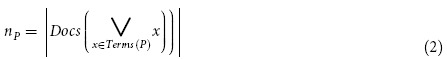



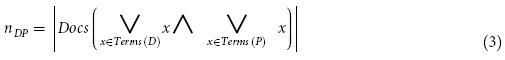
*n*_*tot*_ is the total number of documents in our corpus (5,164,316).

We compute several co-occurrence measures^22^ to determine whether a co-occurrence between a term referring to a phenotype and a term referring to a disease is significant. In particular, we compute the Normalized Pointwise Mutual Information (NPMI), T-Score, Z-Score, and Lexicographer’s Mutual Information (LMI) measures^22^:


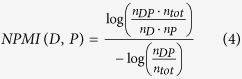



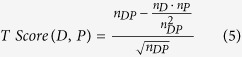



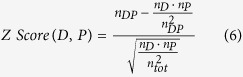



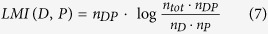


We use NPMI as our primary scoring function for phenotype–disease associations; the other scoring functions are pre-computed and made available for further analysis on our website. Based on a score for a co-occurrence, we can sort phenotype associations for each disease based on decreasing score values. Using this sorted list, we then compute a rank for an association such that the highest-scoring association for a disease is on rank 0. We use this ranking based on the NPMI score to determine a rank-based cut-off; in particular, we set a cut-off based on highest-scoring *p* percent of the associations. Using the rank as cut-off instead of raw score value allows comparison across multiple diseases, as each disease will have a the same number of phenotypes associated, independent of the actual value of the score.

### Semantic similarity

We use the PhenomeNET system to compute the semantic similarity between disease phenotypes and mouse model phenotypes. PhenomeNET^14^ uses a multi-species phenotype ontology that integrates classes from species-specific phenotype ontologies into a single structure in which classes are related based on their formal definitions^20^. For example, the HPO class *Tetralogy of Fallot* (HP:0001636) will become a subclass of the MP classes *ventricular septal defect* (MP:0010402), *overriding aortic valve* (MP:0000273) and *abnormal blood vessel morphology* (MP:0000252), among others, based on the definitions that were developed for the classes in both ontologies. As a consequence of this cross-species integration, it becomes possible to directly compare phenotypes and sets of phenotypes across species using approaches based on semantic similarity^43^. In our current study, we use the integrated phenotype ontology developed by the Monarch Initiative (http://monarchinitiative.org/), downloaded on 22 June 2014.

To compare sets of phenotypes (either associated with a disease, or observed in a mouse model), we use the set-based simGIC measure^44^. The simGIC measure is based on the Jaccard index weighted by information content of a class within the corpus consisting of mouse models and diseases:


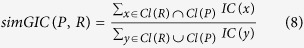


where *Cl*(*X*) is the smallest set containing *X* and which is closed against the superclasses relation (i.e., 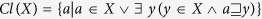). *IC*(*x*) is the information content of a class 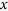 within the corpus of mouse models and diseases (i.e., 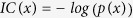).

### Evaluation with gene-disease associations

We evaluate our text-mined disease phenotypes by comparing the semantic similarity between the disease phenotypes and mouse model phenotypes. We assume that semantic similarity over phenotype ontologies (“phenotypic similarity”) is indicative of a causal relation between the mutation underlying the mouse phenotypes and the disease. For this purpose, we compare the results against three curated datasets of known gene-disease associations for heritable diseases. All other associations are treated as negative instances for the purpose of the evaluation. As evaluation datasets, we use three sets of genotype-disease associations: the gene-disease associations from OMIM’s MorbidMap^3^ and the genotype-disease associations from the MGI database^41^. We generate the third evaluation dataset by taking the genotype-disease associations from the MGI database, filtering by single gene knockouts, and merging all phenotypes associated with one gene. As a result, our third dataset consists of gene-disease associations. We refer to the three evaluation datasets as “OMIM”, “MGI” and “MGI (genes)”, respectively.

We use receiver operating curve (ROC) analysis to evaluate and quantify the predictive power of the text-mined disease phenotypes. A ROC curve is a plot of the true positive rate of a classifier as a function of the false positive rate, and the area under the ROC curve (ROCAUC) is a quantitative measure of a classifier’s quality^45^. True positives are the known genotype-disease associations in our evaluation dataset, and false positives are all other genotype-disease associations. In particular, we assume that the genotype-disease associations in our evaluation datasets are exhaustive. Since this assumption is almost certainly incorrect and some of the associations we treat as false positives will in fact be positive instances (i.e., genuine associations between a gene or genotype and a disease), the reported ROC AUC values are a lower boundary of the true performance of our method. We report the ROC AUC values together with an estimate of the 95% confidence interval^46^: we use 
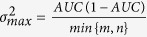
, with *m* and *n* being the number of positive and negative instances in the evaluation dataset, and then use *AUC *± 2*σ* as an estimate of the 95% confidence interval.

### Evaluation with drug-disease associations

The SIDER 2 database^23^ is a resource that contains drugs and their indications which are text-mined from package inserts. SIDER uses UMLS to identify diseases. We use the mappings between DO and UMLS which are distributed as part of the DO to identify the corresponding diseases for drug indications in SIDER. As a result, we identify 65,882 drug–disease associations where drugs refer to entries in the SIDER database and diseases are identified by DO. We then identify diseases that share a drug, i.e., diseases which are treated with the same drug, resulting in 34,986 disease pairs. Using ROC analysis, we then identify the probability of ranking these disease pairs higher than other pairs of diseases.

### Phenotype similarity network

To generate the phenotypic similarity network between diseases, we use disease classes from DO as nodes. We create an undirected weighted edge between two nodes if the similarity between the two associated diseases is in the highest-ranking 0.5% similarity values within our distribution of 38,688,400 similarity values between diseases (*p *≤ 0.005 within this distribution). We remove all self-loops from the network (i.e., edges with a similarity value of 1.0 connecting a node with itself), and finally we remove all remaining nodes with a degree of 0 from the network. Network analysis was performed in Gephi^47^.

In addition to generating the network based on the *p *≤ 0.005 cutoff, we apply the parameter-free clustering algorithm FLAME^24^ to the disease–disease similarity matrix and identify 198 clusters. We compare the clusters against divisions induced by top-level DO classes. For each class *D* from DO, let *T*(*D*) be the set of DO top-level classes of which *D* is a subclass. We then identify the majority top-level class *M* in each cluster 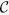, and define purity as:


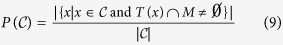


Average purity over multiple clusters is the arithmetic mean of the individual cluster purity values.

The Rand index^25^ compares two clusterings, and we use the Rand index to determine the similarity between the clustering derived by applying the FLAME clustering algorithm and the division of DO classes as induced by DO’s top-level classes. For the clustering 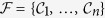 derived by FLAME and the division of disease classes 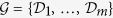 induced by DO, let *x* be the number of pairs of diseases that are in the same set in 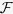 and 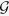, and *y* be the number of pairs of diseases that are in different sets in 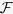 and 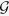. The Rand index (with *n* being the number of disease classes) is defined as:


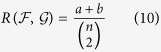


### Interface and visualization

The web interface was written in Groovy (backend) and Javascript (frontend). The disease network was visualized using the Gephi graph visualization tool^47^, and the disease network browser was generated with Gephi’s Sigma-js export plugin (http://blogs.oii.ox.ac.uk/vis/). All graphs are visualized using a force-directed layout.

## Additional Information

**How to cite this article**: Hoehndorf, R. *et al.* Analysis of the human diseasome using phenotype similarity between common, genetic, and infectious diseases. *Sci. Rep.* **5**, 10888; doi: 10.1038/srep10888 (2015).

## Figures and Tables

**Table 1:** Phenotypic homogeneity of disease categories. We compute ROCAUC values for top-level categories in DO. Diseases are ranked based on phenotypic similarity, true positive matches are diseases in the same top-level DO category, and negative matches are diseases in different DO categories.

**Disease category**	**ROCAUC**
bacterial infectious disease (DOID:104)	0.743 ± 0.057
cardiovascular system disease (DOID:1287)	0.720 ± 0.045
disease by infectious agent (DOID:0050117)	0.738 ± 0.035
disease of cellular proliferation (DOID:14566)	0.672 ± 0.021
disease of mental health (DOID:150)	0.783 ± 0.049
disease of metabolism (DOID:0014667)	0.733 ± 0.053
endocrine system disease (DOID:28)	0.711 ± 0.076
fungal infectious disease (DOID:1564)	0.864 ± 0.072
gastrointestinal system disease (DOID:77)	0.718 ± 0.045
genetic disease (DOID:630)	0.656 ± 0.0707
immune system disease (DOID:2914)	0.730 ± 0.046
integumentary system disease (DOID:16)	0.743 ± 0.051
musculoskeletal system disease (DOID:17)	0.704 ± 0.048
nervous system disease (DOID:863)	0.712 ± 0.026
parasitic infectious disease (DOID:1398)	0.729 ± 0.070
physical disorder (DOID:0080015)	0.593 ± 0.113
reproductive system disease (DOID:15)	0.868 ± 0.048
respiratory system disease (DOID:1579)	0.868 ± 0.047
syndrome (DOID:225)	0.613 ± 0.138
thoracic disease (DOID:0060118)	0.659 ± 0.212
urinary system disease (DOID:18)	0.870 ± 0.050
viral infectious disease (DOID:934)	0.745 ± 0.074
